# Remote sensing for land cover mapping across Victoria, Australia – a machine learning application

**DOI:** 10.1038/s41597-025-04900-5

**Published:** 2025-04-03

**Authors:** Sabah Sabaghy, Mohammad Abuzar, Doug Crawford, Andy McAllister, Kathryn Sheffield

**Affiliations:** 1 Agriculture Victoria Research, Victorian Department of Energy, Environment and Climate Action, Bundoora, Victoria 3083 Australia; 2 Agriculture Victoria Research, Victorian Department of Energy, Environment and Climate Action, Ellinbank, Victoria 3821 Australia; 3 Agriculture Victoria Research, Victorian Department of Energy, Environment and Climate Action, Tatura, Victoria 3616 Australia

**Keywords:** Environmental impact, Climate change

## Abstract

This paper presents a detailed land cover mapping study for the state of Victoria in Australia conducted through a machine learning approach (random forest algorithm) using Sentinel-2 imagery. The study uses a hierarchical classification, based on the FAO’s Land Cover Classification Scheme. This paper highlights the importance of spatial random sampling in assessing ground data, and details methods for land cover mapping, remote sensing analysis, calibration as well as validation. The land cover mapping procedure involves the use of a fine-tuned random forest classifier, and an overlaying mask generation technique to improve classification accuracy. The resulting 2021/22 land cover map is accessible through the Victorian Land Use Information System (VLUIS) and has undergone rigorous technical validation with an overall accuracy of 86%. This data set is publicly accessible and regularly released to provide valuable information for a variety of applications, including agricultural policy development, strategic planning, climate change modelling and environmental monitoring.

## Background & Summary

The terms land cover and land use are occasionally used interchangeably, yet there is a subtle difference between them. Land cover is related to the physical attribute of the land surface, while land use relates to how people make use of the land^1^. Changes in land use and land management lead to changes in land cover^2^. Examples of land cover include trees, shrubs, grasslands, bare soil, rocky areas and water bodies^3^. Built areas, plantations and crops are also sometimes included in the definition of land cover. Detailed information on land cover is required for a wide range of applications. Examples include food security, monitoring of extreme weather events (floods and droughts), climate change modelling, hydrological modelling, and monitoring of environmental changes such as vegetation phenology and fire occurrence^4–6^.

Remotely sensed observations (satellite imagery in particular) has been a major source of information that has been used to map the land cover^7^. Modern technology has significantly improved the capabilities of satellites in being able to provide more accurate and detailed information at more frequent time intervals and coverage across greater sections of the electromagnetic spectrum. To effectively use this satellite data, more powerful classification techniques have emerged, predominantly through machine learning based techniques. The random forest algorithm is a machine learning technique that is often used for land cover classification with great success and time efficiency^8–12^.

This work identifies the patterns of land cover across Victoria using the random forest machine learning algorithm alongside Sentinel-2 imagery and field/desktop data. Former land cover mapping across Victoria utilized decision trees based on field data-derived thresholds, applied to the Moderate Resolution Imaging Spectroradiometer (MODIS) imagery^6^. This resulted in a spatial resolution of 250 m for the previous land cover maps in Victoria. However, using Sentinel-2 with its superior spatial resolution (10 m to 20 m) will allow to produce more detailed land-cover maps.

**Fig. 1:**
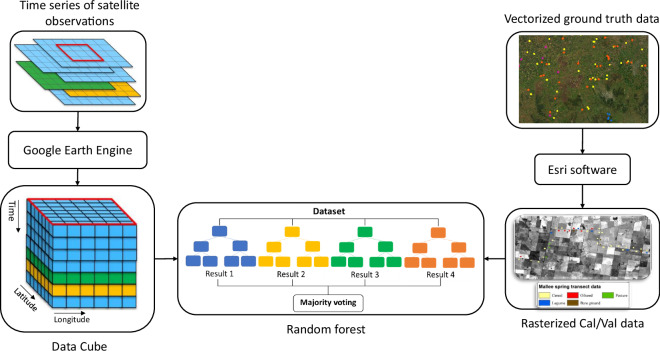
Key components of the land cover mapping procedure.

Figure 1 shows the key components of the land cover mapping process in a schematic diagram. The aim is to adhere to this plan for generating yearly land cover maps throughout Victoria. The annual land cover maps help in tracking changes in land cover over time across the entire state. Moreover, it will enhance our comprehension of agricultural crop rotation trends from year to year and how land cover responds to seasonal and annual climate variations.

## Methods

### Land cover spatial coverage

**Fig. 2:**
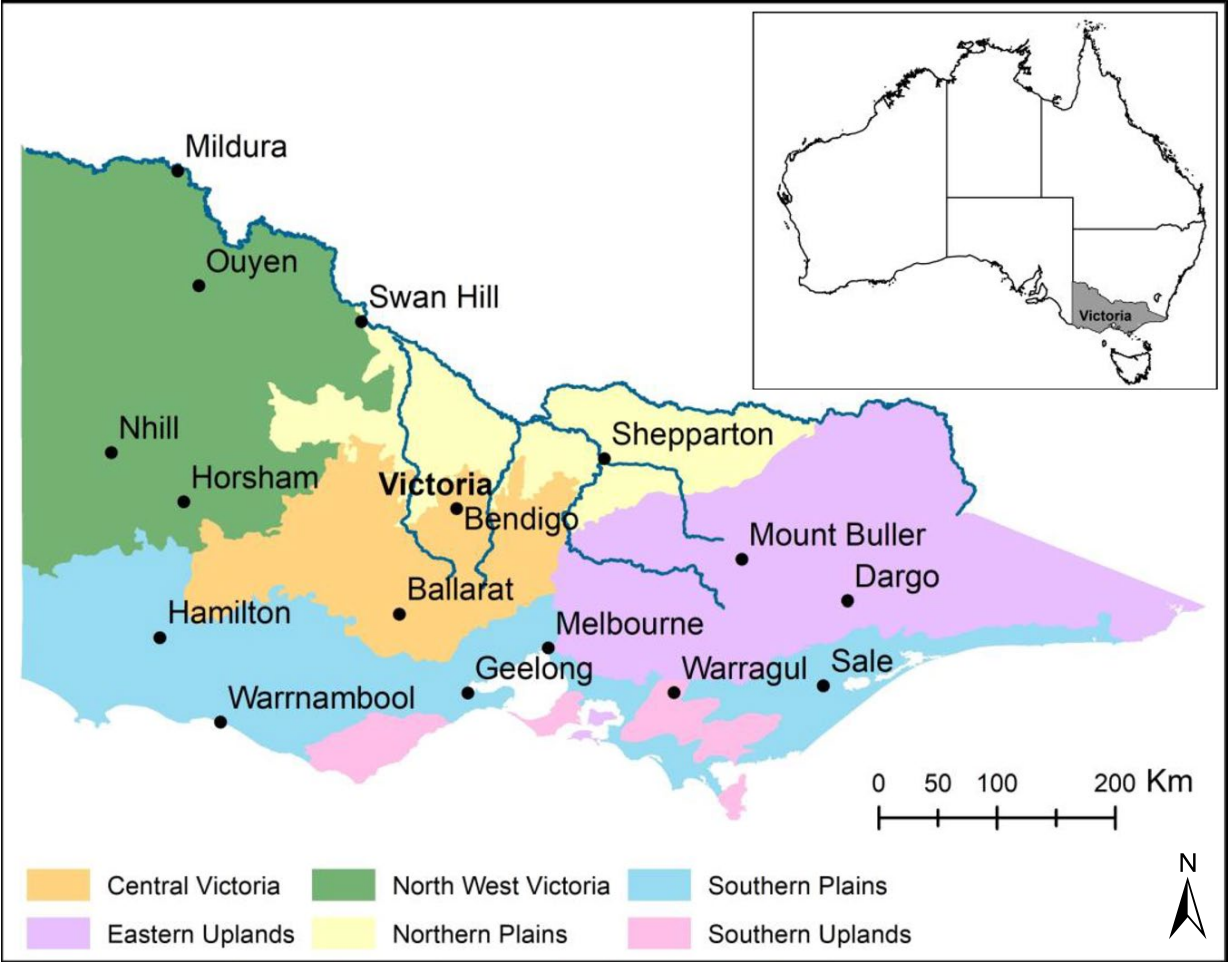
Spatial coverage of Victoria, Australia, highlighting diverse Primary Production Landscape (PPL) Classes. PPL refers to a diverse range of landscapes and terrestrial ecosystems in the Victoria. PPL is explained in detail in the subsection ‘Calibration and validation data’.

The generated land cover map^13^ provides details for Victoria (Fig. 2) in the south-eastern part of Australia. Victoria is approximately 227,100 square km in size and is bordered by New South Wales to the north and South Australia to the west. Victoria exhibits a diverse landscape. Half of the state’s immense landmass is covered by primary production, such as dryland cropping, horticulture, livestock grazing and irrigated agriculture, which includes viticulture and dairy. Tree plantation, native forests, bushland, mines, parks, navigable water, built-up area, and roads also contribute to give Victoria its unique landscape character. Almost 38% of the state is comprised of public land (government-owned). The population, which is predominantly centred on the capital city of Melbourne, is more than 6.5 million people. The climate is diverse, from the semi-arid conditions in the northwest, through alpine land in the east, to a more temperate climate in the south. The rainfall across state varies from less than 300 mm to more than 2,500 mm. Notably, the agricultural regions surrounding Mildura, Horsham, and Warragul have annual rainfall averages varying from 280 mm to 1,022 mm. The Murray River forms part of the northern border of Victoria and various nature features from the north-east alpine plateaus to the western deserts are in Victoria. These varying landscapes contribute to the state’s wide-ranging climate conditions, shaping the diverse experiences of weather across its regions.

### Land cover classification scheme

The FAO Land Cover Classification System (LCCS)^14^ (https://www.fao.org/land-water/land/land-governance/land-resources-planning-toolbox/category/details/en/c/1036361/) was used as a guiding standard for classification purposes. This ensured consistent adherence to class definitions, accuracy evaluations, and reporting methodologies^15^. LCCS provides a hierarchical classification. The hierarchical structure developed for the Victorian land cover has three primary classes (water, vegetated terrestrial, and non-vegetated terrestrial) which are further sub-divided into 4 secondary, 6 tertiary, and 14 quaternary classes that are listed in Table 1.

**Table 1:** VLUIS hierarchical classification and land cover classes.

FAO Level 1	FAO Level 2	FAO Level 3	FAO Level 4	VLUIS land cover
Water	Water	Water	No vegetation	Water
Vegetated	Vegetated-Terrestrial	Vegetated-Terrestrial-Natural	Woody vegetation	Native woody vegetation
			Herbaceous vegetation	Pastures and grassland
		Vegetated-Terrestrial-Cultivated	Woody vegetation	Deciduous fruit and nut trees
				Evergreen fruit and nut trees
				Hardwood tree plantation
				Softwood tree plantation
			Herbaceous vegetation	Cereals
				Legumes
				Oilseeds
				Vegetables and herbs
Non-vegetated	Non-vegetated-Terrestrial	Non-vegetated-Terrestrial	No vegetation	Urban area
				Bare ground and non-photosynthetic vegetation

### Remotely sensed data

Sentinel-2 Level-1C (orthorectified top-of-atmosphere reflectance) plays an important role in land cover mapping^16^ across Victoria. The use of multispectral Sentinel-2 imagery with a spatial resolution of 10 to 20 m^17^, provides valuable insights into the state of the land surface. From April 2021 to March 2022, the Sentinel-2 data was acquired every five days, although the frequency of usable imagery was impacted by cloud cover. This period (April to March) is aligned with Australian national land use datasets to capture key agricultural practices such as winter crops, summer crop rotation and irrigation practices. The satellite images were downloaded from Google Earth Engine, employing a process involving the investigation of inter-band correlation across four contrasting regions and seasons in Victoria. The Sentinel-2 images were processed in VicGRID94 map projection (EPSG 3111).

The relative correlation of bands Blue (B2), Green (B3), Red (B4), Red Edge (B5, B6, B7), Near-Infrared (NIR, B8), Narrow NIR (B8a), and Shortwave Infrared (B11 and B12) was analysed to bring out the significant correlation and help in choosing uncorrelated bands for land cover classification. In particular, the correlation between bands Blue (B2), Green (B3), and Red (B4) has been found to be strong enough that band Green (B3) was chosen because of its strong reflection in a healthy plant and sensitivity to slight change of chlorophyll content^18^. Similarly, bands Red Edge (B6, B7), Near-Infrared (B8), and Narrow NIR (B8a) displayed high inter-correlation, with Narrow NIR (B8a) selected for its distinct characteristics. Narrow NIR (B8a) mirrors the traits of Landsat OLI’s NIR band^19^, exhibiting sensitivity to variations in surface reflectance^20^. It is sensitive to spectral variability of vegetation, thus performing better in identifying temporal and spatial heterogeneity features in vegetation. There has also been testing of the coherence among bands Red Edge (B5) and Shortwave Infrared (B11 and B12) to provide choice in deciding on which band to use for this study. Shortwave Infrared (B11 and B12) were strongly correlated. For better detection of leaf water, Shortwave Infrared (B11), which has shorter wavelength, was chosen. Shorter wavelengths are known to have better sensitivity to leaf water content^21^.

The correlation among four spectral indices was also analysed. These indices, including Enhanced Vegetation Index (EVI)^22^, Normalized Difference Vegetation Index (NDVI)^23^, Normalized Difference Water Index (NDWI)^24^, and Soil-Adjusted Vegetation Index (SAVI)^25^, have proven effective in distinguishing various land cover types^26–28^. Although these indices have been shown to be linked to each other^29^, they were included in the classification procedure to provide more information on how vegetation and surface water are represented spectrally. NDVI is widely used to assess vegetation health and density by measuring the contrast between near-infrared (which is very strongly reflected by vegetation) and red light (which is absorbed by vegetation). NDVI is critical in separating vegetated surfaces from non-vegetated surfaces^30^. The EVI enhances the sensitivity of vegetation detection by optimizing the vegetation signal, particularly in areas of dense canopy^31^. It reduces atmospheric impacts and soil background signal correction and hence is advantageous for vegetation detection in areas of dense forest. While water bodies were not the primary focus of our machine learning-based classification, NDWI was included to improve accuracy. This index is widely used to monitor changes in water resources^29^. Its inclusion helps prevent misclassification of water as vegetation, especially in areas where the two are mixed, e.g., ephemeral water bodies. SAVI is a modified version of NDVI that accounts for the impact of soil brightness in areas with limited vegetation cover using a soil brightness correction factor^25,29^. The correction facilitates more accurate tracking of vegetation in arid and semi-arid regions where there is high soil exposure. The use of these features will help to understand more clearly the complexity of land cover changes in Victoria. Relevant retrieval algorithms for the indices are supplied beneath Table 2.

**Table 2:** List of spectral bands and indices employed for classification.

Bands	Central wavelength (nm)
B3 (Green)	559.8
B5 (Vegetation red edge)	704.1
B8a (Narrow NIR)	864.7
B11 (Shortwave Infrared)	1613.7
EVI^1^	NA
NDVI^2^	NA
NDWI^3^	NA
SAVI^4^	NA

Temporal aggregates of these bands, comprising autumn, winter, spring, and summer medians, were utilized to generate a data cube encompassing 32 bands.

^1^Sentinel-2 EVI = 2.5 * (B8 − B4) / (1 + B8 + 6 * B4 − 7.5 * B2).

^2^NDVI = (NIR − Red) / (NIR + Red) = (B8 − B4) / (B8 + B4).

^3^NDWI = (B3 − B8) / (B3 + B8).

^4^SAVI = ((B8 − B4) / (B8 + B4 + L)) * (1 + L); L is the soil brightness correction factor (commonly 0.5 for moderate vegetation cover).

The second phase of the data processing was creating temporal aggregates of seasonal medians of spectral bands and indices listed in Table 2. It is important to note that all the spectral features listed in this table were harmonized to a 20 m spatial resolution before the data was used for classification purposes. Multi-temporal datasets help reduce classification uncertainty when compared to single datasets^32^. In addition, the relevance of a median of reflectance values extends across a wide time span rather than being confined to a specific time period^33^ and also assists in overcoming issues such as irregular image capture and cloud cover. This can be a significant challenge working in study areas such as Victoria, which are captured by a range of orbits at different time periods.

### Calibration and validation data

Calibration and validation data play a crucial role in the classification process using random forest. Due to field limitations and project scheduling, gathering roadside survey details for the 2021/22 land cover layer was not possible, except for some data obtained within the Mallee Catchment Management Authorities (CMA) for another project. As an alternative, a desktop analysis was conducted by examining trustworthy sources (including Digital Agriculture Services (DAS) Crop raster data^34^, Vicmap plantation data^35^, VLUIS 2016/17 ground data^36^, Department of Energy, Environment and Climate Action (DEECA) land cover “dryland cropping” classification data^37^, and Multi-temporal native vegetation extent for Victoria^33^) and digitized representative samples aligned with the image pixel footprints.

Variability of land cover types across various landscape areas results in uncertainty in the validation process. To effectively reduce this uncertainty, a stratified random sampling approach^6^ was applied, where spatially randomised sampling of ground data was conducted within distinct landscape categories to enable sampling of training data over all spaces, making training data representable across a range of areas. This involved first partitioning landscapes into distinct sub-regions with similar biophysical characteristics^38^. This was accomplished by associating each category of the land cover mapping training data with the Primary Production Landscape (PPL) layer^39^. The PPL layer consists of six unique landscape units (shown in Fig. 2) designed for the detailed exploration of the effects of climate change and variability on agricultural sectors, soil conditions, and management approaches, specifically tailored for Victoria^40^.

According to Thanh Noi and Kappas^41^ findings, significant classification accuracy was attained in both imbalanced and balanced datasets when the training sample size was large enough and adequately represented around 0.25% of the total study area. This study aimed to gather 630 samples (including both calibration and validation data) for each classification category, in line with available resources. Nevertheless, certain classes were consistently underrepresented based on their frequency within the landscape. The data collection process happened after organizing data into sub-regions, from which an equal number of data points were randomly chosen from each PPL cluster. This allocation ensured 500 samples per land cover class for training a random forest model across Victoria, while 130 samples (about 25% of the total digitized data) served as an independent dataset for evaluating the final classified land cover data^13^.

Efforts were made to obtain representative samples at the pixel level of satellite data. This involved visual interpretation, which is a widely accepted method for this purpose^42^. In addition to visual interpretation, an active vegetation analysis was conducted to help with selecting samples associated with active vegetation cover. This phase entailed generating an active vegetation layer, mapped using Sentinel-2 NDVI. In this process, a pixel is classified as ‘active’ if it had a certain species of vegetation exceeding an NDVI threshold at some time during the year. That threshold is NDVI ≥0.4 for crops and the pastures subject to irrigation and careful management^43^. A slightly lower threshold of NDVI ≥0.35 was used for native vegetation and areas with sparse vegetation cover.

**Fig. 3:**
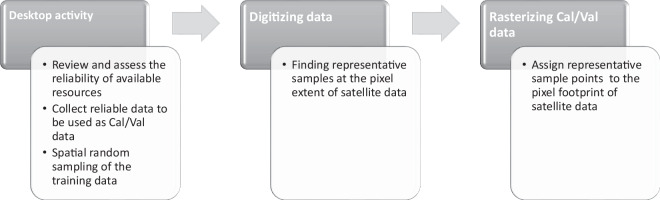
Preparation of calibration and validation (Cal/Val) data.

Training data points were collected in the form of a GIS feature class. Training data was then converted to a raster file which matches the spatial resolution and footprint of the Sentinel-2 data for the use in land cover mapping. The process for creating the training data is shown in Fig. 3.

### Land cover mapping procedure

The random forest of individual decision trees - used for the land cover mapping of Victoria -illustrates a powerful classifier^12^. This classifier is an ensemble learning method, which uses many simple classifiers or decision trees to map land cover types for each individual pixel of Sentinel-2 imagery with 20 m resolution. Within the ensemble, each tree suggests a land cover type for a given pixel. In a voting process, the suggested land cover type that comes up the most is then assigned to the pixel. The power of the random forest algorithm comes from the ability to look at combinations of features and their interactions in addition to looking at individual features on their own. This collaboration between the learners provides a more powerful function. For this reason, assigning classes to image pixels improves in terms of quality, as the pixels get mapped to the exact land cover class based on the random forest paradigm.

The higher the number of classes, the harder it is for the algorithm to efficiently separate and classify each category^11,44,45^. Because of the availability of reliable information about urban and built-up areas and the straightforward application of NDVI and NDWI thresholds for generating water bodies and bare ground information, an approach was employed to reduce the number of classes involved in the classification scheme. This approach involved excluding urban and built-up areas, water bodies, and bare ground classes from the random forest classification scheme. The final version of the land cover mapping was created by ‘overwriting’ these areas, using only the prior information about these classes that were already available. A mask was created using the prior information for the excluded classes - further details on creation of the mask for overlaying appear in the subsection ‘Creation of overlaying mask’. The mask determines the pixels to be excluded from classification by the random forest algorithm. This mask was used to overwrite unclassified pixels and recapture information from existing data on the urban, built-up, water and bare-ground classes into the classified image. The goal here was to improve accuracy by utilising data already present, without contributing to the classifications themselves, and to decrease computation times associated with training and applying the random forest classifier in other areas of the landscape.

Google Earth Engine is a cloud-based computing and data storage platform^46^ that allowed us to filter all the image bands including simple raw spectral data and seasonal vegetation indices. In this way, there was no need to download and store the raw imagery and process it locally as the work was instead completed in the cloud. As explained above, the correlation between Sentinel-2 spectral bands was analysed, leading to the selection of four key bands (Blue (B3), Red Edge (B5), Narrow NIR (B8a) and Shortwave Infrared (B11)). This was followed by the inclusion of four widely used Sentinel-2 indices for land cover mapping. The eight selected spectral bands and indices, listed in Table 2, were used to generate seasonal aggregations for Australia—summer (December to February), autumn (March to May), winter (June to August), and spring (September to November)—resulting in a total of 32 bands for classification. A method was also created to work with Google Earth Engine to extract the image cubes using a tiled format. This has the advantage of decreasing the total computational requirements and processing time by approximately 50%.

**Fig. 4:**
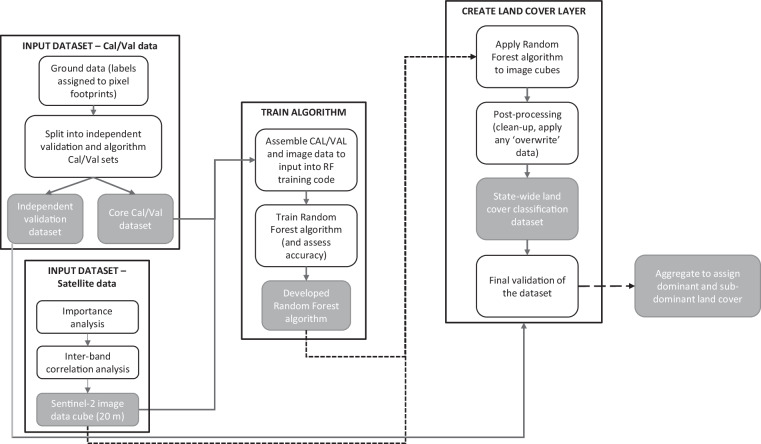
Schematic diagram of the land cover classification approach. Grey boxes highlight key outputs at each stage, while white boxes represent processing steps or intermediate actions contributing to the final classification.

Using the ground truth data and image tile cubes as inputs, a fine-tuned random forest classifier was developed (schematised in Fig. 4). This classifier was developed using the python scikit-learn^47^ random forest implementation, with the optimal hyperparameters determined through the application of RandomizedSearchCV. The final model was configured with the following parameters: 2000 decision trees (n_estimators = 2000), a maximum tree depth of 25 (max_depth = 25), square root selection of features for node splits (max_features = ‘sqrt’), a minimum of 2 samples per leaf (min_samples_leaf = 2), and no bootstrapping (bootstrap = False). The model was initialized with a fixed random state (random_state = 42) to ensure reproducibility. The classifier model was initially developed and tested within the Mallee CMA using land cover calibration/validation data obtained during the Mallee spring transect survey^48^ conducted in September 2021. The Mallee is a region in Victoria, which is characterized by semi-arid to arid landscapes. The region is known for its dry climate, sandy soils, and sparse vegetation. This phase involved evaluating how high a spatial resolution was required to make an effective classification. The spatial resolution of 10 m, expected to provide considerable amounts of spatially detailed information across the entire state, demonstrated no clear benefits in delivering a better classification in terms of accuracy. The higher spatial resolution introduced sources of error due to variations within a single land cover class, leading to increased classification inconsistence. Additionally, the 10 m resolution required significantly higher computational resources for processing, making it less efficient for large-scale applications.

The overall accuracy of the random forest model for the 10 m resolution was 89.7%, with a kappa coefficient of 0.89, while the 20 m resolution achieved a comparable overall accuracy of 89.0% and a kappa coefficient of 0.88. For most land cover classes, accuracy remained similar between the two resolutions. However, certain classes such as softwood plantations, pastures and grasslands, cereals, and legumes showed lower accuracy in the 10 m product compared to the 20 m resolution. For example, softwood tree plantations had F1-score of 0.71 at 10 m but improved to 0.86 at 20 m. Similarly, cereals and legumes saw improved classification accuracy at 20 m resolution. Conversely, in some cases, such as deciduous and evergreen fruit and nut trees, the 10 m resolution performed slightly better, with f1-score values of 0.74 and 0.82, compared to 0.73 and 0.75 for both classes respectively at 20 m. However, these differences were relatively minor.

Overall, sufficient accuracy for classification was found with a 20 m spatial resolution while balancing data storage, computation, and accuracy requirements. Given the minimal accuracy gains at 10 m resolution, along with its higher computational demands and increased classification errors, the 20 m resolution was found to be the more practical choice for large-scale land cover classification.

Following the pilot study in the Mallee, a random forest model was created and fine-tuned throughout Victoria using 70% of a larger training dataset collected across the state. Using the optimised random forest model, the accuracy of both the overall product and specific land cover types were maximised. The optimisation process was an internal evaluation of the random forest model, considering metrics such as user accuracy, producer accuracy, Kappa coefficient, and F1-score. User accuracy measures how closely a pixel value in a category matches its respective ground truth; producer accuracy measures how often object manifest in the ground cover types in each area; and the Kappa coefficient compares the classification against a random assignment, with 1 being a perfect ordinal agreement and 0 being none. The in-machine-learning-used F-score combines producer and user accuracy as a measure of model accuracy ranging from 0 to 1, where 1 is perfect accuracy and 0 is producer or user accuracy being zero. A kappa score near 1 means that the classified image and the ground truth data have a good robust alignment.

### Overlaying mask generation

The overlaying mask contains details regarding three distinct land cover classes, as detailed below:

#### Bare ground

Bare ground has been defined at pixel level using temporal NDVI values derived from Sentinel-2 images during April 2021 to March 2022. A pixel was identified as no vegetation if the NDVI value of that pixel is less than 0.2 but above zero. If that pixel maintains no vegetation status most of the times (≥80%) when observed during the year, it is identified as bare ground. Built-up areas and major urban areas have been excluded from this layer and masked separately.

Validation of the bare ground mask was conducted against the 2021 Digital Earth Australia (DEA) Land Cover Map C3^49,50^ derived from Landsat imagery. The outcomes show that the bare ground class was 1.00 in user accuracy, 0.64 in producer accuracy, and an F1-score of 0.78. Total accuracy for the class was 64.17%. These findings show a high accuracy in detecting bare ground pixels, although the producer accuracy indicates some misclassification meaning that some bare ground areas were missed.

#### Water bodies

Surface water across Victoria was systematically mapped utilizing Sentinel-2 satellite imagery spanning from April 2021 to March 2022. This was achieved via a straightforward but commonly applied algorithm known as the NDWI^24,51^. The efficacy of the threshold-based NDWI algorithm was notably enhanced when combined with a vegetation index such as NDVI^52^. Sentinel-2 images covering the entire Victoria for the entire 12-month period underwent processing on Google Earth Engine to compute both NDVI and NDWI. Identification of water presence at the pixel-level relied on specific criteria: NDVI < 0.2 and NDWI > 0.2. Frequency of water presence was quantified as the number of instances in which the algorithm identified water in a location over the year divided by the total temporal observations and expressed as a percentage. Areas where the frequency of water presence was equal to or exceeded 5% were delineated as surface water. The resulting output materializes as a GRID layer with a 10 m spatial resolution, which was then scaled to 20 m matching the resolution and footprint of the satellite data that was used for mapping the land cover.

The performance of the water bodies mapping was evaluated against the 2021 DEA land cover C3 map. User accuracy was calculated at 0.79, while producer accuracy reached 0.88. These measurements indicate high agreement between the mapped water bodies and the reference data, proving the effectiveness of the NDWI algorithm combined with the vegetation index in correctly detecting water presence across Victoria.

#### Urban and built-up area

This dataset combines information about both urban areas and built-up areas. The urban areas data was collected using the Major Urban Area data^53^, defining the boundaries that are of significance to setting noise limits in areas of commercial, industrial and trade premises. The built-up area data was sourced from Vicmap Lite - Place (Built Up Area) Polygon^54^. This is one of the built-in layers in Vicmap Lite and the built-up area polygon features are included in that layer.

## Data Records

**Fig. 5:**
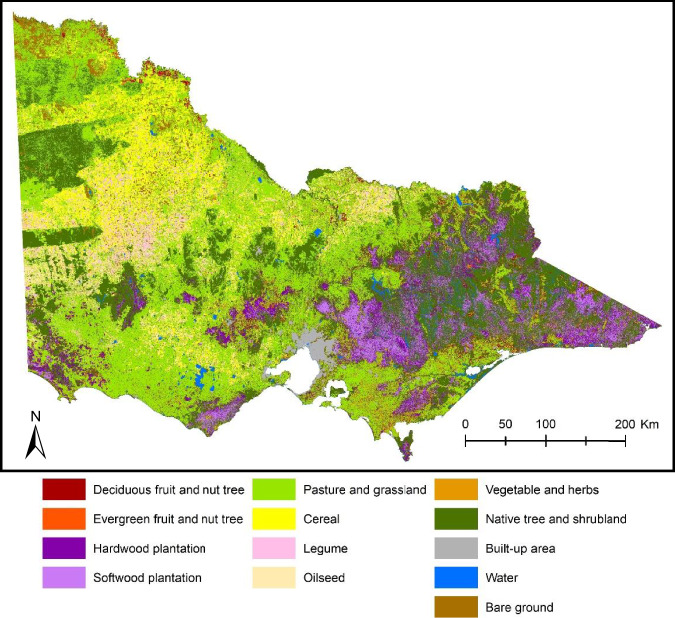
Land cover map across the Victoria state at 20 m spatial resolution for 2021/22.

The 2021/22 land cover map^13^ (shown in Fig. 5) is accessible in the form of a raster dataset. This raster dataset is the foundation of land cover data in the VLUIS vector database, where it was utilized for classifying the dominant land cover category per paddock. VLUIS aims to provide contemporary and detailed spatial information for agricultural policy development, strategic planning, climate change modelling and environmental monitoring.

The 2021/22 land cover map^13^ is publicly available and can be accessed at PANGAEA with a metadata statement document which details its scope and purpose, utility, limitations, as well as spatial and temporal coverage. The intent is to release land cover information on an annual basis with a dataset temporal span of between April and March of the following year. This timing aligns with key agricultural activities in Victoria including winter cropping, summer cropping, and irrigation practices. Each release will include a core VLUIS dataset including land cover, land use and land tenure.

## Technical Validation

**Fig. 6:**
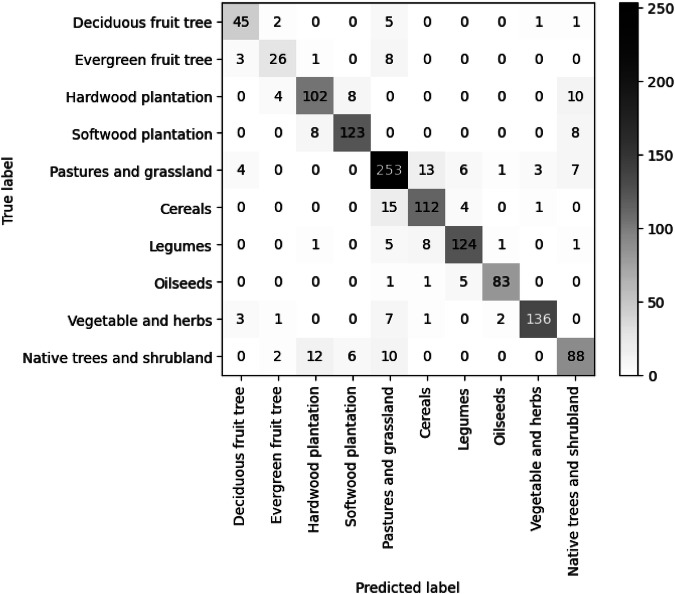
Confusion matrix for the land cover classification.

Results from the internal validation of the trained random forest model are summarised in Table 3. Evaluations were conducted with 30% of the training data. The final best-tuned classification produced a land cover map with an overall accuracy of 86% and a strong kappa value of 0.84. Figure 6 demonstrates the competent classification of land cover classes with minimal misclassification between classes. Notably, there was some confusion observed between the pasture and grasslands category and cereals. Confusion was also observed between native trees/shrubland and hardwood plantation as well as between native trees/shrubland and pastures/grassland among various land cover types.

**Table 3:** Overall accuracy achieved for each land cover category mapped across Victoria.

Land cover type	User accuracy	Producer accuracy	F1-score
Deciduous fruit and nut trees	0.82	0.83	0.83
Evergreen fruit and nut trees	0.74	0.68	0.71
Hardwood tree plantation	0.82	0.82	0.82
Softwood tree plantation	0.90	0.88	0.89
Pastures and grassland	0.83	0.88	0.86
Cereals	0.83	0.85	0.84
Legumes	0.89	0.89	0.89
Oilseeds	0.95	0.92	0.94
Vegetable and herbs	0.96	0.91	0.93
Native shrubland and tree	0.77	0.75	0.76

To ensure that the produced land cover classification map is accurate, the final land cover map was compared against an independent validation dataset, which was 25% of the initial calibration and validation database; this data was withheld entirely from the random forest classification process. Data validation went a step further through multiple manual consistency checks of the compiled VLUIS. This manual consistency check helped to spot errors or signs of concern in the land cover map.

An ArcGIS toolbox was developed for the independent evaluation of the accuracy of land cover data. This toolbox extracts cell values from the VLUIS land cover map when a validation data point is within a pixel from the VLUIS land cover data. Then metrics including the user accuracy, producer accuracy, F1-score, overall accuracy, and kappa coefficient were calculated. The independent evaluation of the land cover map showed an overall accuracy of 83% and a strong kappa value of 0.80. The user accuracy and producer accuracy of each individual land cover class are presented in Table 4.

**Table 4:** Classification accuracy across land cover categories obtained from the independent pixel-based accuracy evaluation of the land cover map.

Land cover type	User accuracy	Producer accuracy	F1-score
Deciduous fruit and nut trees	0.91	0.85	0.88
Evergreen fruit and nut trees	0.83	0.81	0.82
Hardwood plantation	0.71	0.81	0.76
Softwood plantation	0.91	0.88	0.89
Pastures and grassland	0.74	0.86	0.79
Cereals	0.78	0.82	0.8
Legumes	0.89	0.91	0.9
Oilseeds	0.96	0.87	0.91
Vegetable and herbs	0.94	0.87	0.91
Native shrubland and tree	0.84	0.69	0.76

Each category has a classification user accuracy over 0.70 which makes the classified map highly accurate. However, the producer accuracy of native shrubland and tree is the lowest (0.69) compared with the accuracy for other categories considered. Given the similarity of results between the internal random forest classification accuracy assessment and independent land cover map accuracy assessment, we are confident that the random model forest is not over-fitting the data and produces a robust classification.

To further refine the accuracy evaluation, an area adjusted accuracy analysis was conducted, as recommended by Olofsson^55^. Unlike the standard pixel-based accuracy evaluation, which treats all classes equally regardless of their spatial extent, area adjusted accuracy assigns weight to accuracy based on the proportional area of each land cover class. This provides a more balanced and representative evaluation of classification performance. This analysis produced an overall accuracy of 80.86%, closely aligning with the pixel-based assessment.

As shown in Table 5, land cover classes such as pasture and grassland (37.51% of the area) and native trees and shrubland (28.31%) dominate the landscape, while minor classes of vegetables and herbs (1.00%) and deciduous fruit trees (1.95%) are very much less common. The producer accuracy for native trees and shrubland is 0.90, meaning most true occurrences of this class were correctly located (low omission error of 0.09). Its user accuracy is lower at 0.69 meaning most pixels labelled as native trees are not actually native trees (high commission error of 0.31).

**Table 5:** Classification accuracy for each land cover category based on the area adjusted accuracy of the land cover map.

Land cover type	Area proportion	User accuracy	Producer accuracy	F1-score
Deciduous fruit tree	1.95%	0.85	0.47	0.61
Evergreen fruit tree	1.86%	0.81	0.52	0.63
Hardwood plantation	5.84%	0.81	0.40	0.54
Softwood plantation	5.64%	0.88	0.77	0.82
Pasture and grassland	37.51%	0.86	0.86	0.86
Cereals	10.17%	0.82	0.63	0.71
Legumes	4.42%	0.91	0.69	0.78
Oilseeds	3.29%	0.87	0.75	0.81
Vegetable and herbs	1.00%	0.87	0.39	0.54
Native trees and shrubland	28.31%	0.69	0.90	0.78

Conversely, deciduous fruit trees exhibit a high user accuracy (0.85) but a much lower producer accuracy (0.47), suggesting that while most of the mapped pixels in this category are correct (low commission error), many actual deciduous fruit tree pixels were omitted (high omission error of 0.53). This pattern is also seen in vegetables and herbs, where user accuracy is high (0.87), but producer accuracy is only 0.39, meaning many actual occurrences of this class were misclassified as other land types.

By combining the pixel-based accuracy assessment and area adjusted accuracy analysis, we ensure a comprehensive assessment of classification performance. The similarity of outcomes from the two analyses further ensures the consistency of the final land cover map. Although a few slight classification mistakes are present, the general accuracy is good, and the map is appropriate for land cover investigation and decision-making.

## Usage Notes

This paper introduces the approach used for deriving annual land cover maps for Victoria exploiting the random forest algorithm and the Sentinel-2 imagery. The land cover data is fed into VLUIS which is a spatial database of land tenure, land use and land cover information^56^. This type of fundamental data is used in a myriad of applications including strategic planning, food security, resource management, agricultural production monitoring and emergency response. An example of use for the VLUIS data is a part of the Agriculture Climate Spatial Tool (ACST) which supports analyses of agricultural trends, agriculture sector responses to climate and longer-term land cover changes. Land cover data is best to be used in conjunction with land use data.

The spatial resolution of the modelled land cover data is 20 m, improving on the 250 m resolution of previous VLUIS land cover maps. From this information, users will be able to derive land cover information for dominant and sub-dominant land covers per paddock, complementing the VLUIS land use data and enhancing the understanding of land cover variability at a finer scale. These data reflect the VLUIS commitment to provide consistent, repeatable, and resource-efficient land cover information which can be applied to a wide range of research and decision-making requirements.

### Technical notes for data access and processing

The dataset is available for download from PANGAEA in two formats:

- TAB-delimited text format for those who prefer structured text-based data.
- Geo TIFF raster format, which can be accessed by clicking on “View dataset as HTML” in the download section of the PANGAEA webpage. This provides access to a ZIP file containing Geo TIFF raster data and a metadata PDF. This detailed metadata PDF outlines projection information, classification schemes, and attribute definitions.

The Geo TIFF format is compatible with various GIS and remote sensing software such as QGIS, ArcGIS, and ENVI/IDL, while the TAB-delimited text format can be processed in Python, R, or Excel for tabular analysis.

## Data Availability

The custom code associated with this project is openly available on GitHub at https://github.com/SabahSabaghy/RF_Classification_Satellite_Tiles. This repository contains the code necessary to reproduce the data using the same tools. The code is shared under open access terms to ensure transparency and reproducibility of the findings. If you encounter any issues or need further assistance with accessing or using the code, please feel free to reach out.
